# Clinical, biochemical, and genetic spectrum of MADD in a South African cohort: an ICGNMD study

**DOI:** 10.1186/s13023-023-03014-8

**Published:** 2024-01-14

**Authors:** Michelle Bisschoff, Izelle Smuts, Marli Dercksen, Maryke Schoonen, Barend C. Vorster, George van der Watt, Careni Spencer, Kireshnee Naidu, Franclo Henning, Surita Meldau, Robert McFarland, Robert W. Taylor, Krutik Patel, Mahmoud R. Fassad, Jana Vandrovcova, The ICGNMD Consortium, Ronald J. A. Wanders, Francois H. van der Westhuizen

**Affiliations:** 1 Focus area for Human Metabolomics, North-West University, Potchefstroom, South Africa; 2 Department of Paediatrics, Steve Biko Academic Hospital, University of Pretoria, Pretoria, South Africa; 3 Centre for Human Metabolomics, North-West University, Potchefstroom, South Africa; 4 Division of Chemical Pathology, National Health Laboratory Services, University of Cape Town, Cape Town, South Africa; 5 Division of Human Genetics, Department of Medicine, University of Cape Town and Groote Schuur Hospital, Cape Town, South Africa; 6 Division of Neurology, Department of Medicine, Faculty of Medicine and Health Sciences, Stellenbosch University, Stellenbosch, South Africa; 7 Wellcome Centre for Mitochondrial Research, Translational and Clinical Research Institute, Faculty of Medical Sciences, Newcastle University, Newcastle upon Tyne, NE2 4HH UK; 8 NHS Highly Specialised Service for Rare Mitochondrial Disorders, Newcastle Upon Tyne Hospitals NHS Foundation Trust, Newcastle upon Tyne, NE1 4LP UK; 9 Centre for Neuromuscular Diseases, UCL Queen Square Institute of Neurology, London, UK; 10 Department of Clinical Chemistry, Laboratory Genetic Metabolic Diseases, Amsterdam University Medical Centre, University of Amsterdam, Amsterdam, The Netherlands

**Keywords:** Multiple acyl-CoA dehydrogenase deficiency, MADD, Glutaric aciduria type II, *ETFDH*, Riboflavin, South Africa, International Centre for Genomic Medicine in Neuromuscular Diseases, ICGNMD

## Abstract

**Background:**

Multiple acyl-CoA dehydrogenase deficiency (MADD) is an autosomal recessive disorder resulting from pathogenic variants in three distinct genes, with most of the variants occurring in the electron transfer flavoprotein-ubiquinone oxidoreductase gene (*ETFDH)*. Recent evidence of potential founder variants for MADD in the South African (SA) population, initiated this extensive investigation. As part of the International Centre for Genomic Medicine in Neuromuscular Diseases study, we recruited a cohort of patients diagnosed with MADD from academic medical centres across SA over a three-year period. The aim was to extensively profile the clinical, biochemical, and genomic characteristics of MADD in this understudied population.

**Methods:**

Clinical evaluations and whole exome sequencing were conducted on each patient. Metabolic profiling was performed before and after treatment, where possible. The recessive inheritance and phase of the variants were established via segregation analyses using Sanger sequencing. Lastly, the haplotype and allele frequencies were determined for the two main variants in the four largest SA populations.

**Results:**

Twelve unrelated families (ten of White SA and two of mixed ethnicity) with clinically heterogeneous presentations in 14 affected individuals were observed, and five pathogenic *ETFDH* variants were identified. Based on disease severity and treatment response, three distinct groups emerged. The most severe and fatal presentations were associated with the homozygous c.[1067G > A];c.[1067G > A] and compound heterozygous c.[976G > C];c.[1067G > A] genotypes, causing MADD types I and I/II, respectively. These, along with three less severe compound heterozygous genotypes (c.[1067G > A];c.[1448C > T], c.[740G > T];c.[1448C > T], and c.[287dupA*];c.[1448C > T]), resulting in MADD types II/III, presented before the age of five years, depending on the time and maintenance of intervention. By contrast, the homozygous c.[1448C > T];c.[1448C > T] genotype, which causes MADD type III, presented later in life. Except for the type I, I/II and II cases, urinary metabolic markers for MADD improved/normalised following treatment with riboflavin and L-carnitine. Furthermore, genetic analyses of the most frequent variants (c.[1067G > A] and c.[1448C > T]) revealed a shared haplotype in the region of *ETFDH*, with SA population-specific allele frequencies of < 0.00067–0.00084%.

**Conclusions:**

This study reveals the first extensive genotype–phenotype profile of a MADD patient cohort from the diverse and understudied SA population. The pathogenic variants and associated variable phenotypes were characterised, which will enable early screening, genetic counselling, and patient-specific treatment of MADD in this population.

**Supplementary Information:**

The online version contains supplementary material available at 10.1186/s13023-023-03014-8.

## Background

Multiple acyl-CoA dehydrogenase deficiency (MADD; ORPHAcode 26791), also known as glutaric aciduria type II (GAII), is an autosomal recessive disorder that occurs at a global and ethnically variable prevalence of approximately 1 in 200,000 births [1]. It is caused by pathogenic variants (at least 400) in the *ETFA*, *ETFB* and *ETFDH* genes, which encode for the alpha and beta subunits of the electron transfer flavoprotein (ETF) and ETF-ubiquinone oxidoreductase (ETFQO; EC:1.5.5.1), respectively. Collectively, ETF and ETFQO are responsible for re-oxidising reduced mitochondrial flavin adenine dinucleotide (FADH_2_), which in turn sustains mitochondrial fatty acid β-oxidation (FAO), amino acid catabolism and choline metabolism [2, 3].

Clinically, MADD may be divided into three phenotypes. The first and second (MADD types I and II, respectively) are characterised by the neonatal-onset of severe and often fatal symptoms with multi-system involvement (including leukodystrophy, hypotonia, cardiomyopathy, hepatomegaly, and renal abnormalities), as well as hypoglycaemia, hyperammonaemia, and metabolic acidosis (with/without ketosis) [4–6]. MADD types I and II may be distinguished from each other by the presence (type I) or absence (type II) of congenital abnormalities and are caused, at essentially equal frequencies, by pathogenic variants in *ETFA*, *ETFB*, and *ETFDH* [5]. By contrast, the third phenotype (MADD type III) has a later-onset and presents with milder/delayed symptoms which are highly heterogeneous. Such symptoms may include recurrent/intermittent episodes of lethargy and vomiting; muscle, cardiac and/or liver involvement; as well as lipid storage myopathy, hypoglycaemia, metabolic acidosis, and/or hyperammonaemia. MADD type III is reported to be caused mainly by *ETFDH* variants and is also known as riboflavin (Rb)-responsive MADD owing to its amenability to treatment [5, 7]. To estimate the burden of disease, a disease severity scoring system (MADD-DS3; MADD-disease severity 3) may be used [6]. Moreover, disease-severity is often linked to the patient’s response to treatment, which includes a high-caloric diet with fat and protein restriction, the strict avoidance of fasting, and supplementation with L-carnitine and Rb (as first-line treatment) [8, 9].

At present, South Africa (SA) does not have a compulsory newborn screening (NBS) programme. Instead, the biochemical diagnosis of MADD follows (i) a clinical presentation in symptomatic cases, or (ii) an abnormal NBS profile in asymptomatic cases—during which MADD is defined as a secondary condition. Symptomatic patients undergo a thorough metabolic work-up including (i) urinary organic acids, (ii) plasma/serum acylcarnitines, and (iii) plasma/serum amino acids and display a unique metabolic fingerprint as described in a recent review by Mereis et al*.* [3]. As with other inherited metabolic disorders, final confirmation of the diagnosis is obtained by genetic analysis.

In SA, as with most understudied populations, pathogenic variant screening for inherited metabolic disorders is less common. Such screening is, however, offered for a small selection of disorders (including glutaric aciduria type I, isovaleric acidaemia, galactosemia, and MPV17-hepatocerebral mitochondrial DNA depletion syndrome) following the comprehensive description of cohorts in literature. Currently, substantial knowledge of the genotype–phenotype and biochemical profiles, and the subsequent response to treatment are not available for MADD in the different SA populations. Prior to this study, a novel pathogenic *ETFDH* variant (c.[1067G > A], p.[Gly356Glu]) and one previously described pathogenic variant (c.[1448C > T], p.[Pro483Leu]) were reported to result in MADD in the White SA population [10]. In the homozygous state, these variants cause MADD types I and III, respectively, and it was proposed by van der Westhuizen et al. [10] that the prevalence of c.[1067G > A] in this SA population could possibly be the result of a founder effect. In our study, we aimed to address these hypotheses and other limitations by investigating a cohort of 14 recruited MADD patients, under the auspices of the International Centre for Genomic Medicine in Neuromuscular Diseases (ICGNMD). We extensively characterised the clinical phenotype, biochemical profile, and genetics of MADD in SA, and provide knowledge that will contribute to its timely diagnosis, clinical management, and therapeutic intervention.

## Results and discussion

### Cohort

Over a period of three years and using a retrospective and prospective recruitment strategy at three different centres across SA, 12 apparently unrelated families (ten of White SA and two of mixed ethnicity) with 14 clinically affected patients (five males and nine females) were recruited. All patients were born to non-consanguineous parents and disease onset ranged from birth to 41 years, with four deaths recorded (at 9 days, 14 days, 3 months, and 23 years, respectively). Where possible, a consistent clinical re-evaluation was conducted according to ICGNMD guidelines; for most of the cases, biological samples acquired during acute metabolic presentation (n = 12/14) and following therapeutic management (n = 13/14) were collected/available and could be re-analysed (for the remainder, the original data were used, where available).

It should be noted that SA patients of African and Indian ethnicity have previously been diagnosed with MADD on a biochemical level at the Centre for Human Metabolomics [North-West University (NWU)] and the NHLS Inherited Metabolic Disease Molecular Laboratory [University of Cape Town (UCT)]—the two SA facilities where metabolic testing is offered as a main service (unpublished data). However, while all possible efforts were made to include patients of these population groups, they were regrettably lost to follow-up prior to the study’s onset and an opportunity for subsequent genetic confirmation did not present itself. Moreover, no new cases were identified in these population groups during this study. We therefore recognise a potential referral bias within the population groups included. It is our hope that this problem can be addressed by metabolic and genetic NBS in the future.

### Clinical, biochemical, and genetic features

Overall, the SA MADD cohort displayed heterogeneous clinical presentations (Table 1 and Additional file 1), together with the characteristic diagnostic urinary metabolites associated with MADD (Table 2 and Additional file 2). While plasma/serum acylcarnitine and amino acid findings are typically used for diagnosis, this matrix was limited for the majority of the patients (sample collection and analyses were conducted over a 30-year period) and consequently, the levels of these metabolites in the urine are reported instead. The variants identified, their location in ETFQO, and their pathogenicity, according to the American College of Medical Genetics and Genomics (ACMG) criteria, are described in Table 3 [11]. The elucidation of these variants in the SA populations now enable rapid screening methods which were not available before, thereby allowing the timely confirmation of the disorder (in hours) in symptomatic patients. This prompt identification of MADD-related variants can be used to aid the reproductive choices of carrier/affected patients and is especially valuable for prenatal testing and the testing of neonates who may require immediate therapy following birth (i.e., those affected with MADD type I/II). In all recruited cases, a resolved genotype–phenotype result was obtained. Three distinct groups were observed, based on disease severity and response to treatment, as discussed below.

**Table 1:**
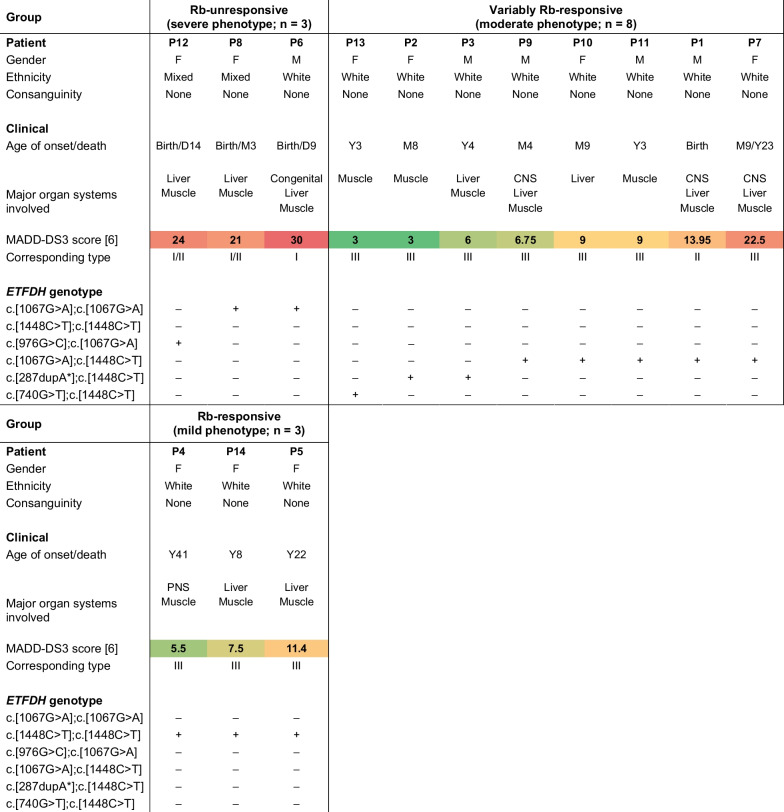
Demographic, clinical, and genetic information of the SA MADD cohort

The MADD-DS3 score [6] increases with disease severity and reflects the clinical symptoms exhibited during decompensation. Consequently, it is affected by the time elapsed (and thus the progression of symptoms) from presentation to successful diagnosis. The MADD-DS3 score is used as a guide only and is not an absolute indication of the degree of pathogenicity of a variant. Red, yellow, and green: high, moderate, and low MADD-DS3 scores within the cohort, respectively

Abbreviations: + Present and validated, *CNS* Central nervous system, *D and number* Age in days, *ETFDH* Electron transfer flavoprotein-ubiquinone oxidoreductase gene (NM_004453.4), *F* Female, *M* Male, *M and number* Age in months, *MADD-DS3* Multiple acyl-CoA dehydrogenase deficiency-disease severity 3, *n* Number of patients, *PNS* Peripheral nervous system, *Rb* Riboflavin, *Y and number* Age in years

**Table 2:**
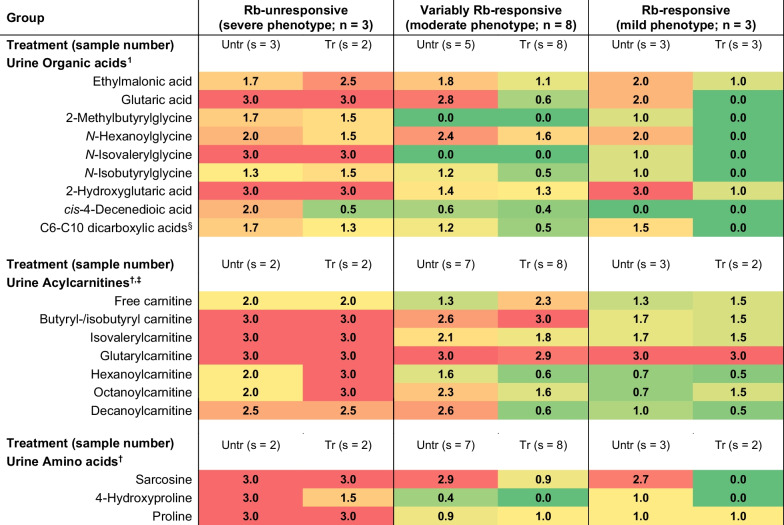
Diagnostic urine MADD metabolites of the SA MADD cohort

^§^The average weighted mean of adipic acid (C6), suberic acid (C8), and sebacic acid (C10) is given

^†^Acylcarnitine and amino acid assessments are typically conducted on plasma/serum, but this matrix was limited due to sample stability (collection ranged over a 30-year period)

^‡^Increased free carnitine is beneficial and promotes fatty acid conjugation and release. Likewise, elevated acylcarnitine conjugates indicate sufficient detoxification

Values represent the number of times a metabolite is increased above the reference range, as a weighted mean of each group (≥ 3: severely elevated; ≥ 2: elevated; > 1: slightly elevated; ≤ 1: normal). Metabolites due to secondary consequences are not reported. Red, yellow, and green: high, moderate, and low values, respectively

Abbreviations: *n* Number of patients, *Rb* riboflavin, *s* Number of available samples, *Tr* Treated; *Untr* Untreated

### Severe, Rb-unresponsive MADD patient group

The first group included three patients (P6, P8 and P12), who presented with the hallmark features of neonatal-onset MADD. Clinically, their symptoms were severe and progressive, and patients were metabolically and phenotypically unresponsive to treatment with Rb. The first case was a male neonate (P6), born prematurely (at 38 weeks) to parents of White SA ethnicity, who presented within the first week of life with acute metabolic decompensation. Key clinical features included congenital cardiac abnormalities (pulmonary stenosis, a patent foramen ovale with a right to left shunt, and a large atrial septum aneurysm) and convulsions with early neonatal death on day 9 of life. Metabolic profiling indicated the typical MADD biochemical fingerprint [12], including dicarboxylic aciduria, increased 2-hydroxyglutaric acid, elevated glycine conjugates (short-, short-branched-, and medium-chain-related), increased disease-associated acylcarnitine conjugates, as well the characteristic increase in sarcosine on the amino acid profile. Both FAO and branched-chain amino acid catabolism were affected, correlating with previous studies on severe MADD cases [6]. The patient displayed a MADD-DS3 score of 30, further supporting a diagnosis of MADD type I. A then novel homozygous variant—c.[1067G > A] (p.[Gly356Glu])—with in silico and structural evidence of pathogenicity (ACMG classification: likely pathogenic) was identified in this neonate [10].

The second patient was a female infant (P8), born at term to parents of mixed ethnicity, who presented at birth with metabolic acidosis, hypotonia and feeding difficulties. Disease-specific metabolic markers were similar to those of P6, and the MADD-DS3 score of 21 was high. The patient succumbed at the age of three months. The homozygous c.[1067G > A] variant was subsequently identified; however, based on the lack of any reported congenital features (absent post-mortem examination), a diagnosis of MADD type I/II was given. The third case was a female neonate (P12), born at term to parents of mixed ethnicity. A urinary organic acid profile typical of MADD was confirmed by the hospital that made the diagnosis (no residual urine collected before or after treatment was available to re-analyse for the purpose of this study). Clinical features included metabolic acidosis, hypoglycaemia, hyperammonaemia, pancytopaenia, acute kidney injury, hyponatremia, and hypocalcaemia, and the patient succumbed at 14 days of age. Based on the absence of any reported congenital features (no post-mortem examination) and the high MADD-DS3 score of 24, a diagnosis of MADD type I/II was given. Whole exome sequencing (WES) and segregation analysis revealed that the patient was compound heterozygous for c.[976G > C];c.[1067G > A] (p.[Gly326Arg];p.[Gly356Glu]). Variant c.[976G > C] is classified as a likely pathogenic variant of unknown significance according to the ACMG criteria and, to our knowledge, has been reported to occur in only one late-onset case of Chinese ethnicity by Xi et al. [13] as compound heterozygous with the common variant, c.[250G > A] (p.[Ala84Thr]; ACMG classification: pathogenic). By contrast, the c.[1067G > A] variant has only been encountered in the SA population to date [10].

Considering the treatment-unresponsive metabolic profile (P6 and P12) and rapid clinical deterioration of patients P6, P8 and P12, it may be inferred that c.[1067G > A] is a highly pathogenic variant. Its presence on both alleles, or its bi-allelic combination with another variant affecting the same protein domain (ubiquinone-binding domain) of ETFQO, appears to lead to insufficient enzymatic compensation for adequate ETFQO activity. While it is evident that L-carnitine supplementation facilitated the formation of disease specific acylcarnitine conjugates, we hypothesise that the homozygous c.[1067G > A];c.[1067G > A] and compound heterozygous c.[976G > C];c.[1067G > A] genotypes result in an ETFQO protein of which the folding cannot be sufficiently rescued/stabilised by Rb treatment.

### Moderate, variably Rb-responsive MADD patient group

The second group included eight patients who presented with moderate, heterogeneous phenotypes, all showing a varying response to treatment. The onset of symptoms was observed in the neonatal period (P1), infancy (P2, P7, P9 and P10), as well as childhood (P3, P11 and P13), and all patients displayed the characteristic clinical features of MADD. These included metabolic decompensation (n = 5), muscle weakness (n = 4), muscle pain (n = 3), hypotonia (n = 5), neck flexor weakness (n = 5), susceptibility to fatigue (n = 2), restrictive ventilatory defect (n = 1), gastrointestinal involvement (n = 6), elevated creatine kinase (CK) (n = 2), recurrent infections (n = 2), lethargy (n = 2), cognitive disability (n = 2), delayed gross motor development (n = 2), migraine/paroxysmal headache (n = 3), seizures (n = 2), coma (n = 3), skeletal involvement (n = 2), and liver dysfunction (n = 4). Uncommon symptoms included ketosis at the time of metabolic crisis (n = 5) and Beevor’s sign (n = 1). Owing to the availability of data, the baseline urine organic acids of only five of the eight patients are reported, and the data of P7 represent the urinary organic acids present upon considerable decompensation near the time of demise.^1^ At first presentation, patients P1, P7 and P9–P11 displayed an increase (to a variable extent) in the diagnostic urine organic acid markers associated with MADD, albeit less pronounced than that of P6, P8 and P12. Most of these patients had increased concentrations of urinary glutaric acid, ethylmalonic acid, dicarboxylic acids, and 2-hydroxyglutaric acid. The excretion of *N*-hexanoylglycine and, to a variable extent, branched-chain-related glycine conjugates were mostly observed. Moreover, all eight patients displayed increased disease-associated urinary acylcarnitines and elevated sarcosine. The biomarker assessment correlated with previous observations in moderate cases where FAO seems to be initially/mostly affected and branched-chain amino acid catabolism is influenced to a lesser extent [6]. It is important to note that the metabolic profiling was greatly dependent on the time of sample collection and that P3 and P9 had received L-carnitine treatment from an early stage in their lives due to the prior diagnosis of a sibling with MADD. Apart from P7, the clinical symptoms, together with most of the urine metabolites, improved upon dietary adjustment in combination with treatment with L-carnitine (P9 and P10), L-carnitine and Rb (P2, P3, P11 and P13), or L-carnitine, Rb, and coenzyme Q10 (P1). Carnitine conjugation indicated that accumulating acyl-CoAs were being detoxified via the carnitine transportation system, which likely explains the less prominent MADD organic acid signature observed. By contrast, the metabolic response to treatment of P7—who succumbed to a stroke at the age of 23 years—was more comparable to that of severe MADD, a finding that correlated with the severity of the clinical presentation as summarised in Table 1 and Additional file 1.

WES and segregation analysis by Sanger sequencing revealed four compound heterozygous variants in this group of White SA-ethnicity patients. These included: (i) c.[740G > T];c.[1448C > T] (p.[Gly247Val];p.[Pro483Leu]) in P13, (ii) c.[287dupA*];c.[1448C > T] (p.[Asp97Glyfs*24];p.[Pro483Leu]) in siblings P2 and P3, and (iii) c.[1067G > A];c.[1448C > T] (p.[Gly356Glu];p.[Pro483Leu]) in P1, P7, P9, P10, and P11. The novel c.[287dupA*] variant affects the third exon of *ETFDH*, leading to a premature stop codon, and is classified as likely pathogenic according to the ACMG criteria. The c.[740G > T] variant shares the same classification as c.[287dupA*], and has been reported only once before as a compound heterozygous variant along with the likely pathogenic c.[389A > T] (p.[Asp130Val]) variant in a late-onset MADD case of Chinese ethnicity [14]. All variants identified in this group encode for highly conserved amino acids.

Once again, the MADD-DS3 scores confirmed that the disease burden increases when the c.[1067G > A] variant is present. This finding is corroborated by the level of steady state ETFQO protein in skin fibroblasts of P5 (c.[1448C > T];c.[1448C > T]) and P9 (c.[1067G > A];c.[1448C > T]), which show a 55% and 73% decrease, respectively, in comparison to fibroblasts from healthy controls (Additional file 3). Similarly, van der Westhuizen et al. [10] reported an 83% decrease in the steady state level of ETFQO in muscle from a patient with a compound heterozygous c.[1067G > A];c.[1448C > T] genotype, when compared to a healthy control. By contrast to the severe, Rb-unresponsive MADD patient group, four of the five patients affected by the c.[1067G > A] variant (P9, P10, P11 and P1) in this group were found to be very amenable to treatment. It is, therefore, reasonable to conclude that when c.[1067G > A] is encountered as a compound heterozygous variant along with a variant which affects a different protein domain of ETFQO (e.g., c.[1448C > T]), sufficient enzymatic compensation occurs which may allow for adequate ETFQO activity. Based on the onset of disease, a diagnosis of either MADD type II (P1) or type III (P2, P3, P7, P9, P10, P11 and P14) was given to the patients in this group.

### Mild, Rb-responsive MADD patient group

The final group included three patients (P4, P5 and P14) of White SA ethnicity, who presented later in life with mild and non-progressive (treatment-related) phenotypes. Clinically, their symptoms were heterogenous, but to some extent characteristic of MADD. The disease presentation included metabolic decompensation (n = 2), muscle weakness (n = 3), neck flexor weakness (n = 2), gastrointestinal involvement as the disease progressed (n = 1), elevated CK (n = 2), lethargy (n = 1), encephalopathy (n = 1), cerebral white matter abnormalities (n = 1), and liver dysfunction (n = 1), with two patients exhibiting ketosis. Initially, statin-induced myositis was suspected in P5 until hepatic features, including lipid deposits on the liver biopsy and raised transaminases prompted a metabolic work-up. P5 and P14 displayed urine metabolites associated with MADD, including dicarboxylic aciduria, raised 2-hydroxyglutaric acid (P5), acylglycine conjugates (with less prominent branched-chain-related conjugates), short- and medium-chain acylcarnitines as well as the presence of sarcosine on the amino acid profile. As indicated earlier in the moderate MADD group, FAO tends to be more affected compared to the branched-chain amino acid catabolism in the less severe cases [6], which correlated with our findings. Literature shows that plasma/serum acylcarnitine profiling is typically most informative when diagnosing a late-onset MADD case, as organic acid profiling may only be remarkable at the time of a metabolic crisis or catabolic status induced by fasting. However, acylcarnitine assessments have been inconclusive in some cases, particularly if the patient has insufficient free carnitine available to promote conjugation [5, 12]. The latter has also led to false negative NBS results in mild MADD cases, as reported by Lin et al. [15]. In our study, P14 showed a free carnitine concentration below the limit of detection and normal butyryl/isobutyryl-, isovaleryl- and glutarylcarnitine levels in plasma/serum (results not shown). Interestingly, this patient presented with severe ketosis, as well as a prominent increase in 2-hydroxyglutaric acid and *cis*-4-decenedioic acid with unremarkable increases in glutaric acid and ethylmalonic acid. These inconsistent urinary organic acid findings corroborate the observations of Goodman et al*.* [16] and support their suggestion to discontinue the use of the term “Glutaric aciduria type II,” as it may be diagnostically misleading. In addition, our data indicate that mild cases of MADD may be missed or incorrectly diagnosed (e.g., as a different fatty acid oxidation disorder) on a biochemical level, depending on the metabolic (i.e., anabolic/catabolic status due to the time of sample collection) and systemic free carnitine status of the patient. Consequently, this study emphasises the metabolite variation within this disease group and advocates for repeat testing if the clinical presentation and routine chemistry are suggestive of MADD.

Following dietary adjustment and treatment with L-carnitine, Rb and coenzyme Q10 (P5 and P14), the clinical and biochemical aberrations of P14 essentially normalised. P4, however, presented with only increased ethylmalonic acid. This patient, the mother of P2 and P3, underwent metabolic testing following the children’s diagnosis and was on L-carnitine treatment at the time of sample collection. Clinical-biochemical improvements, specifically after therapeutic intervention, have been observed in several cases of late-onset MADD, and our clinical and metabolic results (although only three cases were included) strongly correlate with previous investigations [5, 17].

Genetic analyses revealed that all three patients had a homozygous genotype for the known pathogenic c.[1448C > T] (p.[Pro483Leu]) variant. This genotype (c.[1448C > T]; c.[1448C > T]) has been reported in numerous other cases that, similarly to this group, presented with adult-onset, Rb-responsive MADD [10, 17]. The Rb-responsive nature of this variant is further corroborated in a study by Cornelius et al. [18], in which it was shown that ETFQO activity in c.[1448C > T]-modified HEK293 cells could be restored from ~ 45 to ~ 85% (corresponding to an increase of ~ 50 to ~ 80% steady-state ETFQO) when moderately Rb-deficient cultures were treated with a saturating concentration of Rb. Therefore, considering the literature, together with the time of onset, severity and treatment response of this group, a diagnosis of MADD type III was given to P4, P5 and P14, despite moderate MADD-DS3 scores.

### Allele frequency spectrum and haplotypes

To determine the allele frequency of the variants identified, PCR–RFLP analysis was used to screen the two most frequently occurring variants, c.[1067G > A] and c.[1448C > T], in the four largest population groups in SA. The study yielded no homozygous or heterozygous genotypes for any of the variants in any of the population groups assessed so that the allele frequencies of the population groups investigated were calculated as < 0.00067% (African, White SA, and Indian ethnicity) or < 0.00084% (mixed ethnicity) (Table 3). All five variants identified in the cohort were subsequently compared to gnomAD (v2.1.1) [19] and the Human Heredity and Health in Africa (H3Africa) project [20]. Of these variants, only two were recorded on gnomAD, displaying exome allele frequencies of < 0.0001% (c.[740G > T] and c.[1448C > T]); hitherto, none of the variants has been identified in the H3Africa data. The absence of the variants in the above-mentioned population databases, together with the frequency at which they were identified in the SA cohort, indicates with high probability the pathogenicity of these five variants and supports their causative role in MADD.

**Table 3:** *ETFDH* variants identified in the SA MADD cohort

Variant GRCh38.p13	Exon	Protein GRCh38.p13	Protein domain	rsID	ACMG classification	Allele frequencies (%)			
						gnomAD^**†**^		H3Africa	This study
c.[287dupA*]	3/13 (ENSE00003628977)	p.[Asp97Glyfs*24]	FAD-binding	–	Likely pathogenic	AF	–	–	–
						E	–	–	–
						AS + I	–	–	–
						M	–	–	–
c.[740G > T]	7/13 (ENSE00001126657)	p.[Gly247Val]	FAD-binding	rs1384574225	Likely pathogenic	AF	0	–	–
						E	0	–	–
						AS + I	0	–	–
						M	0	–	–
c.[976G > C]	9/13 (ENSE00001126640)	p.[Gly326Arg]	Ubiquinone-binding	–	Uncertain significance	AF	–	–	–
						E	–	–	–
						AS + I	–	–	–
						M	–	–	–
c.[1067G > A]	9/13 (ENSE00001126640)	p.[Gly356Glu]	Ubiquinone-binding	–	Likely pathogenic	AF	–	–	< 0.00067
						E	–	–	< 0.00067
						AS + I	–	–	< 0.00067
						M	–	–	< 0.00084
c.[1448C > T]	11/13 (ENSE00003510567)	p.[Pro483Leu]	Carboxy-terminal	rs377656387	Pathogenic	AF	0	–	< 0.00067
						E	0.00004	–	< 0.00067
						AS + I	0.00003	–	< 0.00067
						M	0	–	< 0.00084

^†^Ethnicities are used as reported on gnomAD: AF: African; AS + I: Asian and Indian; E: European non-Finnish; M: mixed ethnicity

Abbreviations: *ACMG* American College of Medical Genetics and Genomics, *ETFDH* electron transfer flavoprotein-ubiquinone oxidoreductase gene (NM_004453.4), *FAD* flavin adenine dinucleotide, *GRCh* Genome Reference Consortium Human Build, *H3Africa* Human Heredity and Health in Africa, *rsID* reference SNP cluster ID

Haplotyping was performed on those patients from whom sufficient DNA could be obtained. Upon recruitment, patients and their families were invited to self-report their ethnicity and region of birth. Consequently, haplotyping was performed on ten patients of White SA ethnicity (P1–P6, P9–P11 and P13) and one patient of mixed ethnicity (P8) born in various geographical regions across SA (including the central, north-eastern, north-western, south-eastern, and south-western provinces).

**Fig. 1:**
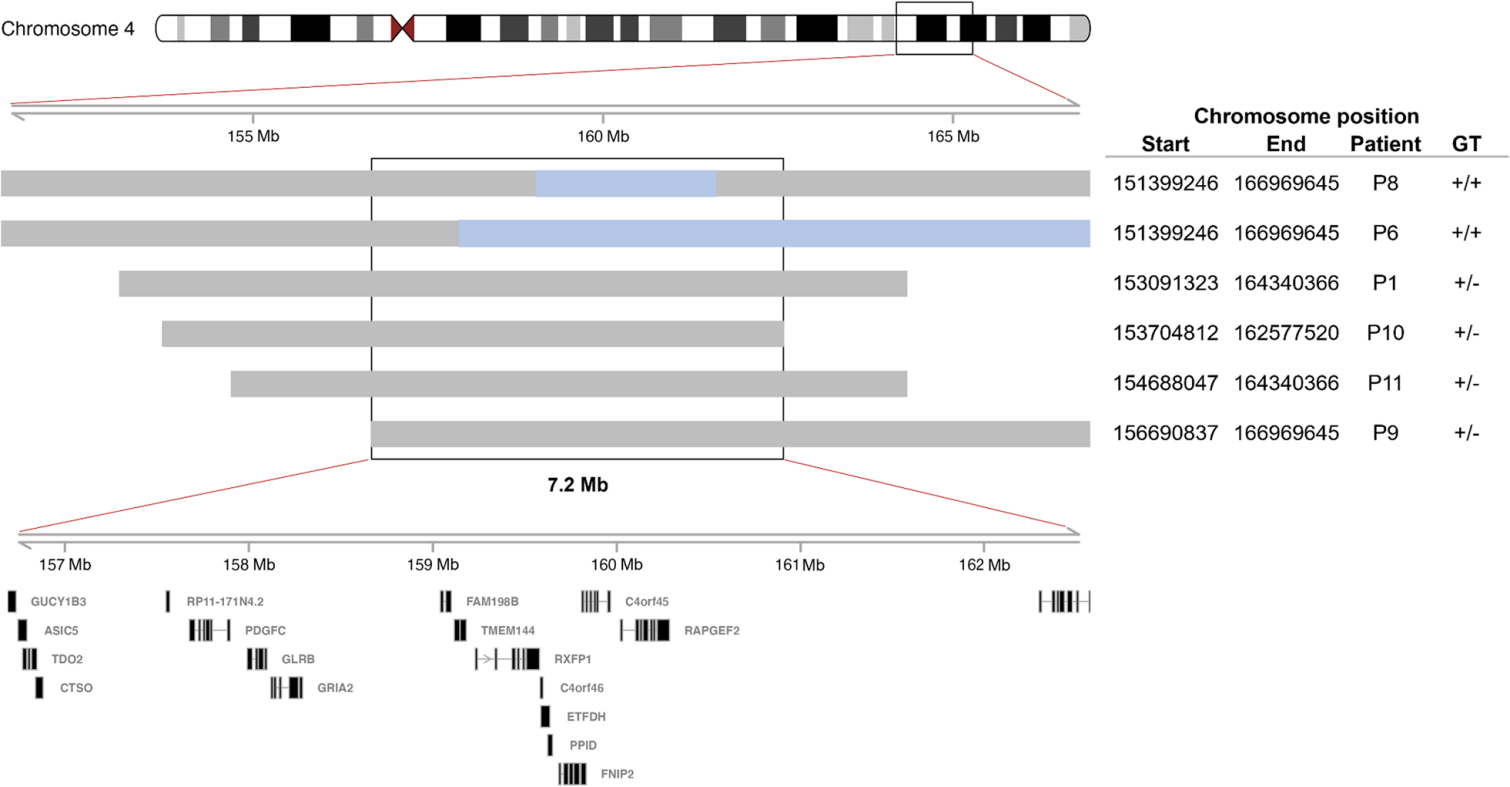
Shared haplotype of patients affected by *ETFDH* c.[1067G > A]. All samples listed show variable lengths of a shared haplotype on a single allele (grey) with a minimal overlapping region of 7.2 Mb, and samples P6 and P8 exhibit homozygosity (c.[1067G > A];c.[1067G > A]) for the shared haplotype (blue). The shared region includes the genes listed. These results indicate a founder for the c.[1067G > A] variant in the White SA population. Abbreviations: *ETFDH* electron transfer flavoprotein-ubiquinone oxidoreductase gene, *GT* genotype

**Fig. 2:**
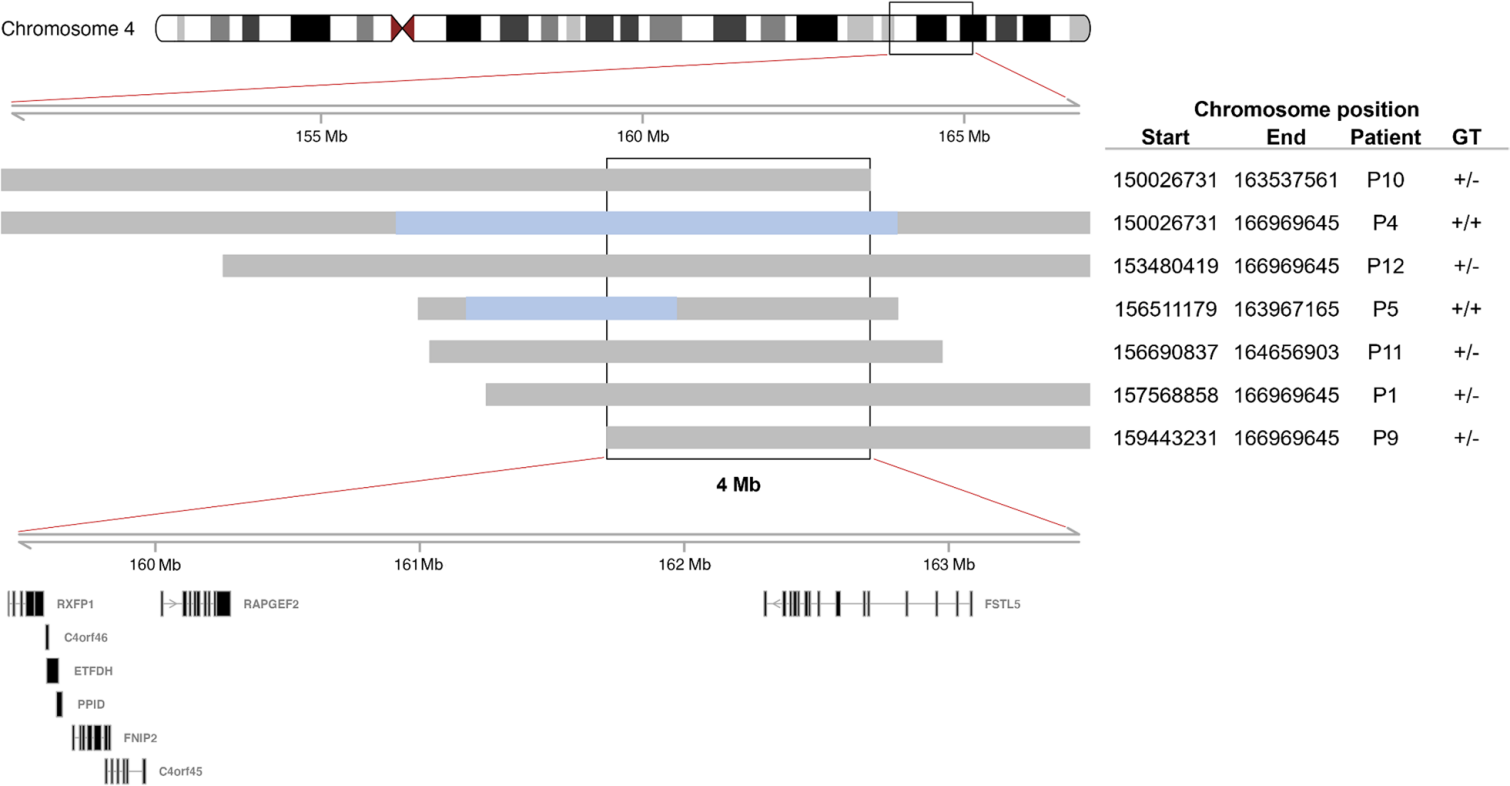
Shared haplotype of patients affected by *ETFDH* c.[1448C > T]. All samples listed show variable lengths of a shared haplotype on a single allele (grey) with a minimal overlapping region of 4 Mb, and samples P4 and P5 exhibit homozygosity (c.[1448C > T];c.[1448C > T]) for the shared haplotype (blue). The shared region includes the genes listed. These results indicate a founder for the c.[1448C > T] variant. Abbreviations: *ETFDH* electron transfer flavoprotein-ubiquinone oxidoreductase gene, *GT* genotype

Apart from the siblings, P2 and P3, and their mother, P4, all patients were found to be unrelated down to the second degree. DNA samples from those patients harbouring the c.[1067G > A] variant (P1, P6, P8 and P9–P11), displayed variable lengths of a shared haplotype on one allele, with a minimal overlapping region of 7.2 Mb (Fig. 1). Of these samples, the two with a homozygous c.[1067G > A];c.[1067G > A] genotype (P6 and P8) exhibited homozygosity for the shared haplotype in the region of *ETFDH*. Similar results were obtained for the DNA samples of those patients harbouring the c.[1448C > T] variant [P1, P4 (including P2 and P3 due to their first-degree relation), P5, P9–P11 and P13], with a minimal overlapping region of 4 Mb (Fig. 2). Again, the two patients who had a homozygous c.[1448C > T];c.[1448C > T] genotype (P4 and P5), displayed homozygosity for the shared haplotype in the *ETFDH* region. These findings suggest the presence of two separate founder haplotypes on which the c.[1067G > A] and c.[1448C > T] variants arose, with that of the former in the White SA population. However, without access to additional control data from the same population(s), it is currently not possible to estimate the haplotype frequency with confidence.

## Conclusion

This study provides the first extensive clinical and biochemical profiles, along with the genetic aetiology, of MADD in the diverse and understudied SA population. Within the cohort investigated, we describe one novel (c.[287dupA*]) and four previously identified causal variants in *ETFDH*, with an autosomal recessive inheritance pattern. In addition, the data support the suspicion that MADD is more prevalent in the White population of SA [10]. We also demonstrate a distinct shared haplotype for each of the two most common variants (c.[1067G > A] and c.[1448C > T]), suggesting the existence of a founder for each, with that of c.[1067G > A] likely having arisen in the White SA population. Furthermore, we show that, depending on the variant(s) involved, it is possible to anticipate the clinical progression and treatment response of the patient, which underscores the need for genetic confirmation in SA. It is our belief, that this genotype–phenotype correlation of MADD in the SA population will assist physicians—who rarely encounter this disorder—to recognise and treat MADD in a more efficient manner. In addition, our study provides background for the subsequent genetic counselling of families and patient-specific treatment of local MADD cases. Altogether, the data reported support a policy of including MADD in the NBS program of SA.

## Methods

### Cohort selection and sampling

Following informed consent/assent, the patients in this study were enrolled as part of the ICGNMD study with ethical approval numbers 19/LO/1796 (HRA and HCRW), NWU-00966-19-A1 and NWU-00966-19-A1-01 (NWU), 296/2019 (University of Pretoria; UP), B19/01/002 (Stellenbosch University; SU), and 605/2020 (UCT).

Probands and their affected/unaffected first-degree relatives were recruited either retrospectively or prospectively, based on a clinical and metabolic diagnosis of MADD, via one of three SA academic, state-funded hospitals: Steve Biko Academic Hospital, Tygerberg Hospital, and Red Cross War Memorial Children's Hospital. For (i) genetic, (ii) protein, and (iii) metabolic analyses, the following samples were obtained: (i) whole blood, saliva (living patients), or urine (deceased patients from whom no blood or saliva was available), (ii) primary skin fibroblasts (P5 and P9), as well as (iii) urine collected during the first metabolic presentation (P1, P4–P6, P8–P12 and P14)/during metabolic decompensation (P7), and after therapeutic management (P1–P11, P13 and P14).

### Clinical and biochemical investigations

Patients were extensively evaluated by paediatric or adult neurologists according to protocols set forth by the ICGNMD. This included obtaining relevant demographic information, family history, medical history, and current treatment (Table 1). Moreover, deep phenotypic data (Additional file 1) were collected during a comprehensive clinical re-assessment of each proband.

SDS-PAGE and Western blot analysis of primary skin fibroblasts, established from two adult patients (P5 and P9) and two healthy controls (matched in age, gender, and ethnicity to the patients), were performed to evaluate the steady state level of ETFQO. Immunoblotting was conducted using primary antibodies at 1:1,000 directed against ETFQO (ab131376; Abcam, Cambridge, UK) and β-actin (as housekeeping gene; ab6276; Abcam), and an HRP-conjugated secondary antibody at 1:10,000 (ab97023; Abcam). Findings are shown in Additional file 3.

For targeted metabolic analyses, urine organic acids and their glycine conjugates were extracted, derivatised, and analysed using the 7890A gas chromatography system coupled to the 5977A MSD mass spectrometer (Agilent Technologies, California, USA) as described by Erasmus et al. [21] and refined by Reinecke et al. [22]. Data acquisition was facilitated with the 5977 MassHunter Data Acquisition software (B.07.04.2260; Agilent Software); Automated Mass spectral Deconvolution and Identification System software (AMDIS v2.73; National Institute for Standards and Technology) was used to identify the component peaks and perform spectral deconvolution. Underivatised amino acids in urine were analysed using validated liquid chromatography-triple quadrupole mass spectrometry and the MassChrom^®^ Amino Acid Analysis kit (75111; Chromsystems, Gräfelfing, Germany). Samples were prepared via the Microlab STAR Liquid Handling System (Hamilton, Nevada, USA) and analysed on the Infinity 1290 II liquid chromatography system coupled to the 6470 Triple Quadrupole liquid chromatography-mass spectrometer (Agilent Technologies). For urine acylcarnitine analysis, electrospray ionisation-tandem mass spectrometry was performed using the 1290 Infinity II liquid chromatography system coupled to the 6410 Triple Quadrupole liquid chromatography-mass spectrometer (Agilent Technologies) as described by Pitt et al. [23]. A standard mixture of stable acylcarnitine isotopes was used for quantification [24]. Amino acid and acylcarnitine data were acquired and quantified using MassHunter Data Acquisition software and MassHunter QQQ Quantitative software (v10.00; Agilent Software), respectively. All reported processed data are shown in Additional file 2.

### Genetic analyses

To identify potential pathogenic variants in the proband DNA, WES was performed at Macrogen Europe (Amsterdam, the Netherlands) using the NovaSeq 6000 HiSeq platform (Illumina Inc., San Diego, USA) and the SureSelect Human All Exon V6 panel (5190-8865; Agilent Technologies). Reads (50 × average depth coverage) were aligned to the Genome Reference Consortium Human Build (GRCh) 38.p13, and variants were annotated according to GRCh38 using the Ensembl Variant Effect Predictor (v108) [25]. High-quality variants were filtered by applying the Genomics’ England PanelApp, “Rhabdomyolysis and metabolic muscle disorders_1.57” panel [26]; the pathogenicity of potential disease-causing variants was evaluated and classified as such, according to the ACMG guidelines [11]. All potential pathogenic variants identified were confirmed and segregation analysis in family members was conducted via Sanger sequencing.

### Allele frequency and haplotyping

To estimate the allele (carrier) frequency of the two most encountered variants in the cohort (c.[1067G > A] and c.[1448C > T]), PCR–RFLP analysis was performed as previously described [10] on newborn dried blood spot (DBS) samples of the four largest SA population groups—African, White SA, Indian, and mixed ethnicity. To this end, a total of 2,844 anonymised and randomised DBS samples per variant per population group were used: 594 representatives of mixed ethnicity as well as 750 representatives of each of the following populations: African, White SA and Indian ethnicity. The samples represented an equal distribution of healthy males and females and were kindly provided by the NBS laboratory at the NWU Centre for Human Metabolomics.

Next, we investigated whether these two variants arose due to founder mutations by determining the haplotype(s) of the affected individuals using a GSA v3.0 array (Illumina Inc.). Sample processing was performed at UCL Genomics (UCL Great Ormond Street Institute of Child Health, London, UK), according to the manufacturer's instructions; the resulting raw IDAT files were processed using Genome Studio (v2.0.5; Illumina Inc.) and converted to PLINK (v1.9) format [27]. Samples and variants with a call rate below 90% were excluded from further analysis. Thereafter, samples were compared to determine familial relations and phased using Eagle (v2.4.1) [28]. Finally, haplotype sharing between each sample was determined with Germline2 software (v1.0) [29] and shared regions were visualised using R (v4.2.2).

### Supplementary Information

**Additional file 1**. Additional Clinical Information.

**Additional file 2**. Additional Metabolic Information.

**Additional file 3**. Additional Structural Information.

## Data Availability

Previous data and samples were made available by the Centre for Human Metabolomics (NWU), SU, and UCT. New samples were collected with the help of paediatric and adult neurologists via Steve Biko Academic Hospital, Tygerberg Hospital, and Red Cross War Memorial Children’s Hospital. The datasets generated and/or analysed during the current study are not publicly available due to the data sharing policy of the ICGNMD study, but are available from the corresponding author on reasonable request.

## Abbreviations

ACMG: American College of Medical Genetics and Genomics
AMDIS: Automated Mass spectral Deconvolution and Identification System
CK: Creatine kinase
CoA: Coenzyme A
DBS: Dried blood spot
DNA: Deoxyribonucleic acid
EC: Enzyme Commission number
ETF: Electron transfer flavoprotein
*ETFA*: Electron transfer flavoprotein subunit alpha (gene)
*ETFB*: Electron transfer flavoprotein subunit beta (gene)
*ETFDH*: Electron transfer flavoprotein-ubiquinone oxidoreductase (gene)
ETFQO: Electron transfer flavoprotein-ubiquinone oxidoreductase
FADH_2_: Flavin adenine dinucleotide (reduced)
FAO: Mitochondrial fatty acid β-oxidation
GAII: Glutaric aciduria type II
gnomAD: Genome Aggregation Database
GRCh: Genome Reference Consortium Human Build
H3Africa: Human Heredity & Health in Africa
HCRW: Health and Care Research Wales
HEK293: Human embryonic kidney 293 cell line
HRA: Health Research Authority
HRP: Horse radish peroxidase
ICGNMD: International Centre for Genomic Medicine in Neuromuscular Diseases
MADD: Multiple acyl-CoA dehydrogenase deficiency
MADD-DS3: Multiple acyl-CoA dehydrogenase deficiency-disease severity 3
Mb: Megabase(s)
MPV17: Mitochondrial inner membrane protein MPV17
MRC: Medical Research Council
n: Number of patients
NBS: Newborn screening
NHLS: National Health Laboratory Service
NHS: National Health Service
NIHR: National Institute for Health and Care Research
NRF: National Research Foundation
NWU: North-West University
NWU-HREC: North-West University-Health Research Ethics Committee
P and number: Patient
PCR-RFLP: Polymerase chain reaction-restriction fragment length
Rb: Riboflavin
SA: South Africa(n)
SAMRC: South African Medical Research Council
SDS-PAGE: Sodium dodecyl sulfate-polyacrylamide gel electrophoresis
SU: Stellenbosch University
UCL: University College London
UCT: University of Cape Town
UK: United Kingdom
UP: University of Pretoria
USA: United States of America
WES: Whole exome sequencing

## Footnotes

1 P2 and P3 (siblings) were diagnosed prior to 1995 (before the storage of electronic records at the contributing diagnostic facility) and did not decompensate following the onset of treatment. Although P7 was diagnosed at the same time, the patient decompensated considerably near the time of her demise. The urinary organic acid data collected during this period of decompensation, while the patient was on Rb and L-carnitine, are reported.
