# Supplementary material for: Clinical, biochemical, and genetic spectrum of MADD in a South African cohort: an ICGNMD study
Source: Orphanet J Rare Dis. 2024 Jan 14;19:15. doi: 10.1186/s13023-023-03014-8 (PMC10789041; doi:10.1186/s13023-023-03014-8)
Supplement: Supplementary file 1 — Additional file 1. Additional Clinical Information. [file 13023_2023_3014_MOESM1_ESM.docx]

**Additional file 1: Additional Clinical Information**

**Table 1** MADD-DS3 domains

| **Group** | **Rb-unresponsive (severe phenotype; n = 3)** | | | **Variably Rb-responsive  (moderate phenotype; n = 8)** | | | | | | | | **Rb-responsive (mild phenotype;  n = 3)** | | |
| --- | --- | --- | --- | --- | --- | --- | --- | --- | --- | --- | --- | --- | --- | --- |
| **Patient** | **P12** | **P8** | **P6** | **P13** | **P2** | **P3** | **P9** | **P10** | **P11** | **P1** | **P7** | **P4** | **P14** | **P5** |
| **MADD-DS3 domains** [1] |  |  |  |  |  |  |  |  |  |  |  |  |  |  |
| Age at onset | 3 | 3 | 3 | 0 | 0 | 0 | 0 | 0 | 0 | 3 | 0 | 0 | 0 | 0 |
| Congenital anomalies | 0 | 0 | 6 | 0 | 0 | 0 | 0 | 0 | 0 | 0 | 0 | 0 | 0 | 0 |
| Cardiac | 0 | 0 | 0 | 0 | 0 | 0 | 0 | 0 | 0 | 0 | 0 | 0 | 0 | 0 |
| CNS | 0 | 0 | 0 | 0 | 0 | 0 | 2.25 | 0 | 0 | 2.25 | 4.5 | 0 | 0 | 0 |
| PNS | 0 | 0 | 0 | 0 | 0 | 0 | 0 | 0 | 0 | 0 | 0 | 1.5 | 0 | 0 |
| Liver | 6 | 6 | 6 | 0 | 0 | 3 | 1.5 | 6 | 0 | 4.5 | 6 | 0 | 4.5 | 6 |
| Muscle | 6 | 3 | 6 | 3 | 3 | 3 | 3 | 0 | 3 | 1.2 | 3 | 4 | 3 | 2.4 |
| Patient-reported outcome | 9 | 9 | 9 | 0 | 0 | 0 | 0 | 3 | 6 | 3 | 9 | 0 | 0 | 3 |
| **Total MADD-DS3 score** | **24** | **21** | **30** | **3** | **3** | **6** | **6.75** | **9** | **9** | **13.95** | **22.5** | **5.5** | **7.5** | **11.4** |

Abbreviations: CNS: central nervous system; MADD-DS3: multiple acyl-CoA dehydrogenase deficiency-disease severity 3; n: number of patients; PNS: peripheral nervous system; Rb: riboflavin.

**Table 2** Clinical symptoms applicable to MADD-DS3 domains

| **Group** | **Rb-unresponsive  (severe phenotype;  n = 3)** | | | **Variably Rb-responsive  (moderate phenotype; n = 8)** | | | | | | | | **Rb-responsive  (mild phenotype;  n = 3)** | | |
| --- | --- | --- | --- | --- | --- | --- | --- | --- | --- | --- | --- | --- | --- | --- |
| **Patient** | **P12** | **P8** | **P6** | **P13** | **P2** | **P3** | **P9** | **P10** | **P11** | **P1** | **P7** | **P4** | **P14** | **P5** |
| **MADD-DS3 domains** [1] |  |  |  |  |  |  |  |  |  |  |  |  |  |  |
| Congenital anomalies |  |  |  |  |  |  |  |  |  |  |  |  |  |  |
| *Hydronephrosis* | U | U | + | – | – | – | – | – | – | – | – | – | – | – |
| Cardiac |  |  |  |  |  |  |  |  |  |  |  |  |  |  |
| *Patent foramen ovale* | U | U | + | – | – | – | – | – | – | – | – | – | – | – |
| *Pulmonary valve stenosis* | U | U | + | – | – | – | – | – | – | – | – | – | – | – |
| CNS |  |  |  |  |  |  |  |  |  |  |  |  |  |  |
| *Epilepsia partialis continua (resolved)* | – | – | – | – | – | + | – | – | – | – | – | – | – | – |
| *Delayed gross motor development* | – | – | – | – | + | – | – | – | – | – | + | – | – | – |
| *Cerebral white matter abnormality* | – | – | – | – | – | – | – | – | – | – | – | – | – | + |
| *Migraine/paroxysmal headache* | – | – | – | – | + | – | – | – | – | + | + | – | – | – |
| *Learning disabilities* | – | – | – | – | – | – | – | – | – | + | – | – | – | – |
| *Intellectual disabilities* | – | – | – | – | – | – | + | – | – | – | – | – | – | – |
| *Encephalopathy* | – | – | – | – | – | – | – | – | – | – | – | – | + | – |
| *Seizures* | – | – | + | – | – | + | – | – | – | – | + | – | – | – |
| *Drooling* | – | – | – | – | – | – | – | – | – | + | – | – | – | – |
| *Stroke* | – | – | – | – | – | – | – | – | – | – | + | – | – | – |
| *Coma* | – | – | – | – | – | – | – | + | + | – | + | – | – | – |
| *Lethargy* | – | – | – | – | + | + | – | – | – | – | – | + | – | – |
| Liver |  |  |  |  |  |  |  |  |  |  |  |  |  |  |
| *Increased liver enzymes* | – | – | + | – | – | – | – | + | – | – | + | – | – | – |
| *Acute hepatic failure* | – | – | – | – | – | – | – | – | – | – | + | – | – | + |
| *Hepatic steatosis* | – | – | – | – | – | – | – | – | – | + | + | – | – | + |
| *Hepatomegaly* | – | – | – | – | + | – | – | + | – | – | + | – | – | – |
| *Hepatosplenomegaly* | – | – | – | – | – | – | – | – | – | – | – | – | – | + |

**Table 2** *(cont.)*

| **Group** | **Rb-unresponsive (severe phenotype; n = 3)** | | | **Variably Rb-responsive  (moderate phenotype; n = 8)** | | | | | | | | **Rb-responsive (mild phenotype; n = 3)** | | |
| --- | --- | --- | --- | --- | --- | --- | --- | --- | --- | --- | --- | --- | --- | --- |
| **Patient** | **P12** | **P8** | **P6** | **P13** | **P2** | **P3** | **P9** | **P10** | **P11** | **P1** | **P7** | **P4** | **P14** | **P5** |
| **MADD-DS3 domains** [1] |  |  |  |  |  |  |  |  |  |  |  |  |  |  |
| Neuromuscular |  |  |  |  |  |  |  |  |  |  |  |  |  |  |
| *Beevor’s sign* | – | – | – | + | – | – | – | – | – | – | – | – | – | – |
| *Elevated CK* | – | – | – | – | – | – | – | – | + | – | + | + | – | + |
| *Facial weakness* | – | – | – | + | – | – | – | – | – | – | – | – | – | – |
| *Neck muscle weakness* | – | – | – | + | + | + | – | – | + | – | + | – | + | + |
| *Susceptibility to fatigue* | – | – | – | – | + | + | – | – | – | – | – | – | – | – |
| *DM weakness lower limbs* | – | – | – | + | + | – | – | – | – | – | – | – | – | – |
| *DM weakness upper limbs* | – | – | – | – | + | – | – | – | – | – | – | + | – | – |
| *PM weakness upper limbs* | – | – | – | + | + | – | – | – | – | – | – | + | + | – |
| *PM weakness lower limbs* | – | – | – | + | + | – | – | – | – | – | – | + | + | – |
| *Weakness* | – | – | – | – | + | – | – | – | – | – | + | + | – | + |
| *Hypotonia* | – | + | – | – | + | + | + | – | – | + | + | – | – | – |
| *Myopathy* | – | – | – | – | + | – | – | – | – | + | + | + | – | + |
| *Pain/myalgia* | – | – | – | – | – | + | – | + | – | + | – | – | – | – |

Abbreviations: +: reported; CK: creatine kinase; CNS: central nervous system; DM: distal muscle; MADD-DS3: multiple acyl-CoA dehydrogenase deficiency-disease severity 3; n: number of patients; PM: proximal muscle; Rb: riboflavin; U: unknown.

**Table 3** Additional clinical symptoms

| **Group** | **Rb-unresponsive (severe phenotype; n = 3)** | | | **Variably Rb-responsive  (moderate phenotype; n = 8)** | | | | | | | | **Rb-responsive (mild phenotype;  n = 3)** | | |
| --- | --- | --- | --- | --- | --- | --- | --- | --- | --- | --- | --- | --- | --- | --- |
| **Patient** | **P12** | **P8** | **P6** | **P13** | **P2** | **P3** | **P9** | **P10** | **P11** | **P1** | **P7** | **P4** | **P14** | **P5** |
| Metabolic |  |  |  |  |  |  |  |  |  |  |  |  |  |  |
| *Hyperammonaemia* | + | + | + | – | – | – | – | – | – | – | + | – | + | + |
| *Metabolic acidosis* | + | + | + | – | – | – | + | – | – | + | – | – | – | – |
| *Aminoaciduria* | – | – | + | – | – | – | – | – | – | – | – | – | – | – |
| *Hypoglycaemia* | + | – | + | – | – | – | – | + | + | + | + | – | + | + |
| *Lactic acidosis* | – | – | + | – | – | – | – | – | – | – | – | – | – | – |
| *Ketosis* | – | – | – | – | + | + | + | + | – | – | + | – | + | + |
| Gastrointestinal |  |  |  |  |  |  |  |  |  |  |  |  |  |  |
| *Chronic constipation* | – | – | – | – | – | – | – | + | – | + | – | – | – | – |
| *Abdominal pain* | – | – | – | – | + | + | – | – | – | – | – | – | – | – |
| *Diarrhoea* | – | – | – | – | – | – | + | – | – | – | – | – | – | – |
| *Vomiting* | – | + | – | – | + | + | – | – | + | – | – | – | + | – |
| Kidney |  |  |  |  |  |  |  |  |  |  |  |  |  |  |
| *Acute kidney injury* | + | – | – | – | – | – | – | – | – | – | – | – | – | – |
| Skeletal |  |  |  |  |  |  |  |  |  |  |  |  |  |  |
| *Lumbar hyperlordosis* | – | – | – | + | – | – | – | – | – | – | – | – | – | – |
| *Scapular winging* | – | – | – | + | – | – | – | – | – | – | – | – | – | – |
| *Osteopaenia* | – | – | – | – | – | – | – | – | – | + | – | – | – | – |
| Other |  |  |  |  |  |  |  |  |  |  |  |  |  |  |
| *Intra-uterine growth restriction* | – | – | + | – | – | – | – | – | – | – | – | – | – | – |
| *Restrictive ventilatory defect* | – | – | – | + | – | – | – | – | – | – | – | – | – | – |
| *Obstructive sleep apnoea* | – | – | – | – | – | – | – | – | – | – | – | – | – | – |
| *Recurrent infections* | – | – | – | – | – | – | – | + | + | – | – | – | – | – |
| *New-onset pancytopaenia* | + | – | + | – | – | – | – | – | – | – | – | – | – | – |
| *Astigmatism* | – | – | – | – | – | – | – | + | – | – | – | – | – | – |

Abbreviations: +: reported; n: number of patients; Rb: riboflavin.

**References:**

[1] van Rijt WJ, Ferdinandusse S, Giannopoulos P, Ruiter JPN, de Boer L, Bosch AM, *et al*. Prediction of disease severity in multiple acyl-CoA dehydrogenase deficiency: a retrospective and laboratory cohort study. *J Inherit Metab Dis.* 2019;42:878–89.
