# Supplementary material for: Clinical, biochemical, and genetic spectrum of MADD in a South African cohort: an ICGNMD study
Source: Orphanet J Rare Dis. 2024 Jan 14;19:15. doi: 10.1186/s13023-023-03014-8 (PMC10789041; doi:10.1186/s13023-023-03014-8)
Supplement: Supplementary file 2 — Additional file 2. Additional Metabolic Information. [file 13023_2023_3014_MOESM2_ESM.docx]

**Additional file 2: Additional Metabolic Information**

**Table 1** Urine Organic acids

| **Status** | **Patient** | **Gender** | **Age** | **Upper reference value^†^** | | **EMA** | **GA** | **2-MBGly** | **HGly** | **iVGly** | **iBGly** | **2-HGA** | **c4DC8** | **DC6** | **DC8** | **DC10** |
| --- | --- | --- | --- | --- | --- | --- | --- | --- | --- | --- | --- | --- | --- | --- | --- | --- |
| **-** | **-** | **M + F** | **-** | **1:** | **Birth-M12** | **10.97** | **4.80** | **2.00** | **1.20** | **10.00** | **3.50** | **-** | **15.80** | **56.23** | **38.40** | **119.31** |
| **-** | **-** | **M + F** | **-** | **2:** | **<Y5** | **24.69** | **8.91** | **2.00** | **1.20** | **10.00** | **3.50** | **-** | **6.17** | **60.34** | **19.20** | **2.74** |
| **-** | **-** | **M + F** | **-** | **3:** | **>Y5; <Y15** | **13.71** | **6.17** | **2.00** | **1.20** | **10.00** | **3.50** | **-** | **3.40** | **9.60** | **17.14** | **3.43** |
| **-** | **-** | **M + F** | **-** | **4:** | **>Y15** | **6.86** | **5.49** | **2.00** | **2.00** | **10.00** | **3.80** | **-** | **0.00** | **61.71** | **5.49** | **-** |
| **-** | **-** | **M + F** | **-** | **5:** | **Birth-M4** | **-** | **-** | **-** | **-** | **-** | **-** | **67.00** | **-** | **-** | **-** | **-** |
| **-** | **-** | **M + F** | **-** | **6:** | **M4-Y2** | **-** | **-** | **-** | **-** | **-** | **-** | **49.00** | **-** | **-** | **-** | **-** |
| **-** | **-** | **M + F** | **-** | **7:** | **Y2-Y10** | **-** | **-** | **-** | **-** | **-** | **-** | **35.00** | **-** | **-** | **-** | **-** |
| **-** | **-** | **M + F** | **-** | **8:** | **>Y10** | **-** | **-** | **-** | **-** | **-** | **-** | **16.00** | **-** | **-** | **-** | **-** |
| Untr | P1 | M | M1 D14 | 1 + 5 | | 36.61 | 36.93 | 1.24 | 29.92 | 0.96 | - | 117.53 | 8.32 | 93.16 | 63.19 | 38.71 |
| Untr | P2 | F | n.d | n.d | | n.d | n.d | n.d | n.d | n.d | n.d | n.d | n.d | n.d | n.d | n.d |
| Untr | P3 | M | n.d | n.d | | n.d | n.d | n.d | n.d | n.d | n.d | n.d | n.d | n.d | n.d | n.d |
| Untr | P4 | F | Y41 M9 | 4 + 8 | | 26.62 | 2.37 | 0.47 | 1.18 | 1.34 | - | 50.80 | 1.35 | 21.61 | 1.29 | - |
| Untr | P5 | F | Y36 M6 | 4 + 8 | | 154.35 | 675.58 | 30.02 | 43.05 | 151.77 | 30.52 | 312.03 | 47.77 | 400.34 | - | 3.03 |
| Untr | P6 | M | D3 | 1 + 5 | | 200.27 | 1370.1 | 7.05 | 40.40 | 444.85 | 27.19 | 1611.9 | 51.59 | 677.00 | 21.06 | 7.69 |
| Untr | P7 | F | Y23 M5 | 4 + 8 | | 0.16 | 86.49 | 0.36 | 10.63 | 4.76 | 16.93 | 54.31 | 21.29 | 126.97 | 1.58 | 1.42 |
| Untr | P8 | F | D6 | 1 + 5 | | - | 2412.2 | 4.33 | 2.25 | 157.98 | 5.52 | 342.16 | 9.82 | 232.80 | 27.53 | 1.09 |
| Untr | P9 | M | M4 | 1 + 6 | | 129.77 | 29.39 | - | 22.98 | - | 2.98 | 75.26 | - | 189.01 | 56.18 | - |
| Untr | P10 | F | Y7 M10 | 3 + 7 | | 25.72 | 215.96 | 1.13 | 44.83 | 8.61 | 10.37 | 95.29 | 79.77 | 393.00 | 77.39 | 42.13 |
| Untr | P11 | M | Y2 M6 | 2 + 7 | | 51.55 | 21.57 | - | - | 4.84 | 3.95 | 31.65 | 1.28 | 55.89 | 11.82 | 1.09 |
| Untr | P12 | F | n.d | n.d | | n.d | n.d | n.d | n.d | n.d | n.d | n.d | n.d | n.d | n.d | n.d |
| Untr | P13 | F | n.d | n.d | | n.d | n.d | n.d | n.d | n.d | n.d | n.d | n.d | n.d | n.d | n.d |
| Untr | P14 | F | Y26 M4 | 4 + 8 | | 5.10 | 23.08 | - | 6.41 | 0.41 | 1.13 | 102.89 | 111.87 | 483.98 | 59.28 | 23.35 |

**Table 1** *(cont.)*

| **Status** | **Patient** | **Gender** | **Age** | **Upper reference value^†^** | **EMA** | **GA** | **2-MBGly** | **HGly** | **iVGly** | **iBGly** | **2-HGA** | **c4DC8** | **DC6** | **DC8** | **DC10** |
| --- | --- | --- | --- | --- | --- | --- | --- | --- | --- | --- | --- | --- | --- | --- | --- |
| Tr | P1 | M | Y11 M5 | 3 + 8 | 13.16 | 1.40 | - | - | 0.64 | 0.67 | 28.46 | 0.57 | 1.38 | 5.18 | - |
| Tr | P2 | F | Y29 M9 | 4 + 8 | 14.80 | 1.56 | - | 7.36 | 0.81 | 2.90 | 26.06 | 0.38 | 7.07 | 3.02 | - |
| Tr | P3 | M | Y25 M8 | 4 + 8 | 21.28 | 1.49 | - | - | 0.52 | 0.64 | 20.96 | - | 2.93 | 1.06 | - |
| Tr | P4 | F | Y45 M1 | 4 + 8 | 5.69 | 1.80 | - | - | 0.15 | 0.08 | 63.07 | - | 4.01 | 1.36 | - |
| Tr | P5 | F | Y40 M2 | 4 + 8 | 19.59 | 1.29 | - | - | 0.03 | - | 13.26 | - | 2.53 | 1.33 | 0.95 |
| Tr | P6 | M | D11 | 1 + 5 | 32.69 | 2915.5 | - | - | 62.67 | 2.10 | 1165.9 | - | - | 6.59 | - |
| Tr | P7 | F | Y22 M1 | 4 + 8 | - | 93.25 | 0.36 | 11.62 | 1.92 | 13.95 | 118.33 | 159.17 | 614.30 | 86.04 | 17.10 |
| Tr | P8 | F | D22 | 1 + 5 | 1053.4 | 2739.6 | 15.03 | 185.63 | 904.67 | 105.58 | 1441.2 | 21.48 | 615.20 | 128.52 | 342.49 |
| Tr | P9 | M | Y18 M9 | 4 + 8 | 40.01 | 8.91 | 0.25 | 33.57 | 2.60 | 7.10 | 58.26 | 17.10 | 87.39 | 24.09 | 7.35 |
| Tr | P10 | F | Y9 | 3 + 7 | 13.07 | 9.99 | 0.20 | - | - | 0.14 | 14.27 | - | 3.97 | 3.65 | - |
| Tr | P11 | M | Y8 M7 | 3 + 7 | 13.15 | 4.52 | 0.37 | 3.40 | 0.74 | 1.81 | 67.05 | 11.02 | 15.94 | 10.01 | 1.6939 |
| Tr | P12 | F | n.d | n.d | n.d | n.d | n.d | n.d | n.d | n.d | n.d | n.d | n.d | n.d | n.d |
| Tr | P13 | F | Y41 M2 | 4 + 8 | 11.27 | 0.94 | - | 4.63 | 0.32 | 1.29 | 5.81 | 0.89 | 3.28 | 2.64 | - |
| Tr | P14 | F | Y26 M8 | 4 + 8 | 7.13 | 0.87 | - | 0.85 | 0.21 | 0.37 | 13.39 | 3.28 | 3.30 | 3.64 | 2.21 |

^†^ Applied reference range is dependent on age. In-house reference ranges were used.

Values are given in mmol/mol creatinine. Abbreviations: 2-HGA: 2-hydroxyglutaric acid; 2-MBGly: 2-methylbutyrylglycine; c4DC8: *cis*-4-decenedioic acid; D and number: age in days; DC6: adipic acid; DC8: suberic acid; DC10: sebacic acid; EMA: ethylmalonic acid; F: female; GA: glutaric acid; HGly: *N*-hexanoylglycine; iBGly: *N*-isobutyrylglycine; iVGly: *N*-isovalerylglycine; M: male; M and number: age in months; n.d: not determined; Tr; treated; Untr: untreated; Y and number: age in years.

**Table 2** Urine Acylcarnitines

| **Status** | **Patient** | **Gender** | **Age** | **Upper reference value^†^ [1]** | | **C0** | **C4** | **C5** | **C5DC** | **C6** | **C8** | **C10** |
| --- | --- | --- | --- | --- | --- | --- | --- | --- | --- | --- | --- | --- |
| **-** | **-** | **M + F** | **-** | **1:** | **Newborn** | **4.89** | **0.84** | **0.38** | **0.63** | **0.23** | **0.2** | **0.1** |
| **-** | **-** | **M + F** | **-** | **2:** | **Y1-Y5** | **13.93** | **0.19** | **0.25** | **0.04** | **0.13** | **0.15** | **0.08** |
| **-** | **-** | **M + F** | **-** | **3:** | **Y6-Y17** | **4.74** | **0.77** | **0.29** | **0.46** | **0.11** | **0.15** | **0.09** |
| **-** | **-** | **M + F** | **-** | **4:** | **≥Y18** | **3.77** | **0.62** | **0.24** | **0.51** | **0.09** | **0.14** | **0.07** |
| Untr | P1 | M | Y1 M9 | 2 | | 456.78 | 14.15 | 4.12 | 2.85 | 0.22 | 0.60 | 0.22 |
| Untr | P2 | F | Y1 M5 | 2 | | 7.71 | 0.44 | 0.43 | 1.95 | 0.18 | 0.27 | 0.34 |
| Untr | P3 | M | D42 | 1 | | 100.68 | 8.19 | 3.78 | 8.47 | 0.47 | 1.30 | 0.64 |
| Untr | P4 | F | Y41 M9 | 4 | | 24.94 | 5.75 | 1.57 | 3.06 | 0.06 | 0.21 | 0.09 |
| Untr | P5 | F | Y36 M7 | 4 | | 5.73 | 0.73 | 0.54 | 5.61 | 0.19 | 0.18 | 0.13 |
| Untr | P6 | M | D3 | 1 | | 26.80 | 38.67 | 61.07 | 17.32 | 1.24 | 1.32 | 0.98 |
| Untr | P7 | F | Y3 M2 | 2 | | 8.54 | 1.69 | 0.89 | 6.72 | 0.35 | 0.71 | 0.67 |
| Untr | P8 | F | 9D | 1 | | 5.71 | 52.03 | 29.15 | 7.89 | 0.23 | 0.39 | 0.26 |
| Untr | P9 | M | Y1 M9 | 2 | | 6.85 | 5.82 | 2.58 | 2.79 | 2.28 | 0.00 | 0.37 |
| Untr | P10 | F | Y7 M11 | 3 | | 31.79 | 1.44 | 0.26 | 1.95 | 0.16 | 0.57 | 0.21 |
| Untr | P11 | M | Y2 M8 | 2 | | 6.61 | 7.20 | 0.66 | 1.93 | 0.16 | 0.24 | 0.18 |
| Untr | P12 | F | n.d | n.d | | n.d | n.d | n.d | n.d | n.d | n.d | n.d |
| Untr | P13 | F | n.d | n.d | | n.d | n.d | n.d | n.d | n.d | n.d | n.d |
| Untr | P14 | F | Y26 M4 | 4 | | 0.94 | 0.65 | 0.09 | 2.02 | 0.04 | 0.11 | 0.08 |
| Tr | P1 | M | Y11 M5 | 3 | | 1.11 | 2.44 | 0.23 | 1.49 | 0.07 | 0.07 | 0.04 |
| Tr | P2 | F | Y29 M9 | 4 | | 21.01 | 5.01 | 0.50 | 2.74 | 0.10 | 0.59 | 0.13 |
| Tr | P3 | M | Y25 M8 | 4 | | 46.34 | 6.30 | 2.14 | 2.49 | 0.14 | 0.55 | 0.08 |
| Tr | P4 | F | n.d | n.d | | n.d | n.d | n.d | n.d | n.d | n.d | n.d |
| Tr | P5 | F | Y40 M2 | 4 | | 1.06 | 0.22 | 0.05 | 2.33 | 0.04 | 0.09 | 0.05 |
| Tr | P6 | M | D11 | 1 | | 1202.20 | 623.42 | 2193.77 | 15.02 | 13.48 | 18.63 | 12.68 |
| Tr | P7 | F | Y13 M4 | 3 | | 46.21 | 4.69 | 0.85 | 4.60 | 0.11 | 0.37 | 0.14 |

**Table 2** *(cont.)*

| **Status** | **Patient** | **Gender** | **Age** | **Upper reference value^†^ [1]** | **C0** | **C4** | **C5** | **C5DC** | **C6** | **C8** | **C10** |
| --- | --- | --- | --- | --- | --- | --- | --- | --- | --- | --- | --- |
| Tr | P8 | F | D22 | 1 | 8.91 | 24.15 | 15.22 | 4.24 | 1.79 | 1.63 | 0.27 |
| Tr | P9 | M | Y18 M9 | 4 | 8.09 | 9.34 | 0.51 | 2.10 | 0.06 | 0.25 | 0.05 |
| Tr | P10 | F | Y9 | 3 | 21.42 | 4.29 | 0.70 | 1.12 | 0.06 | 0.23 | 0.08 |
| Tr | P11 | M | Y8 M7 | 3 | 436.34 | 37.05 | 10.89 | 7.04 | 0.25 | 3.86 | 0.24 |
| Tr | P12 | F | n.d | n.d | n.d | n.d | n.d | n.d | n.d | n.d | n.d |
| Tr | P13 | F | Y41 M2 | 4 | 6.66 | 2.75 | 0.18 | 1.75 | 0.05 | 0.10 | 0.06 |
| Tr | P14 | F | Y26 M8 | 4 | 147.42 | 7.64 | 2.56 | 3.74 | 0.13 | 0.69 | 0.08 |

^†^ Applied reference range is dependent on age.

Values are given in mmol/mol creatinine. Abbreviations: C0: free carnitine; C4: butyryl-/isobutyrylcarnitine; C5: isovalerylcarnitine; C5DC: glutarylcarnitine; C6: hexanoylcarnitine; C8: octanoyalcarnitine; C10: decanoyalcarnitine; D and number: age in days; F: female; M: male; M and number: age in months; n.d: not determined; Tr: treated; Untr: untreated; Y and number: age in years.

**Table 3** Urine Amino acids

| **Status** | **Patient** | **Gender** | **Age** | **Reference range^†^** | **Sarcosine** | **4-OH-Pro** | **Pro** |
| --- | --- | --- | --- | --- | --- | --- | --- |
| **-** | **-** | **M** | **-** | **1** | **0-8.5** | **20-320** | **21-213** |
| **-** | **-** | **F** | **-** | **2** | **0-8.5** | **20-320** | **21-213** |
| **-** | **-** | **M** | **-** | **3** | **0-8.5** | **0-143** | **0-130** |
| **-** | **-** | **F** | **-** | **4** | **0-1.4** | **0-13** | **0-13** |
| **-** | **-** | **M** | **-** | **5** | **0-1.4** | **0-13** | **0-13** |
| **-** | **-** | **M** | **-** | **6** | **0-1.4** | **0-13** | **0-9** |
| **-** | **-** | **F** | **-** | **7** | **0-1** | **0-13** | **0-9** |
| **-** | **-** | **M** | **-** | **8** | **-** | **0-6** | **64-236** |
| **-** | **-** | **M** | **-** | **9** | **-** | **0-5** | **43-173** |
| Untr | P1 | M | Y1 M9 | 5 | 7.94 | 3.83 | 12.43 |
| Untr | P2 | F | Y1 M5 | 4 | 25.86 | 7.06 | 32.48 |
| Untr | P3 | M | D42 | 3 | 92.71 | 240.39 | 94.27 |
| Untr | P4 | F | Y41 M9 | 7 | 9.84 | 2.31 | 4.29 |
| Untr | P5 | F | Y36 M7 | 7 | 60.09 | 106.42 | 41.70 |
| Untr | P6 | M | D3 | 1 | 67.25 | 1004.76 | 1149.35 |
| Untr | P7 | F | Y3 M2 | 7 | 10.98 | 7.86 | 14.64 |
| Untr | P8 | F | D9 | 2 | 106.99 | 1407.46 | 1674.14 |
| Untr | P9 | M | Y1 M9 | 5 | 5.14 | 34.65 | 132.79 |
| Untr | P10 | F | Y7 M11 | 7 | 2.63 | 1.68 | 4.00 |
| Untr | P11 | M | Y2 M8 | 6 | 6.83 | 2.03 | 6.89 |
| Untr | P12 | F | n.d | n.d | n.d | n.d | n.d |
| Untr | P13 | F | n.d | n.d | n.d | n.d | n.d |
| Untr | P14 | F | Y26 M4 | 7 | 2.90 | 0.27 | 1.32 |

**Table 3 *(cont.)***

| **Status** | **Patient** | **Gender** | **Age** | **Reference range^†^** | **Sarcosine** | **4-OH-Pro** | **Pro** |
| --- | --- | --- | --- | --- | --- | --- | --- |
| Tr | P1 | M | Y11 M5 | 8 | 0.70 | 0.75 | 24.64 |
| Tr | P2 | F | Y29 M9 | 7 | 1.16 | 0.29 | 5.75 |
| Tr | P3 | M | Y25 M8 | 9 | 2.58 | 0.87 | 32.60 |
| Tr | P4 | F | n.d | n.d | n.d | n.d | n.d |
| Tr | P5 | F | Y40 M2 | 7 | 0.35 | 0.23 | 9.20 |
| Tr | P6 | M | D11 | 1 | 93.18 | 510.92 | 4118.11 |
| Tr | P7 | F | Y13 M4 | 7 | 0.57 | 0.67 | 1.53 |
| Tr | P8 | F | D22 | 2 | 37.5 | 873.2 | 1505.58 |
| Tr | P9 | M | Y18 M9 | 9 | 3.15 | 0.31 | 10.89 |
| Tr | P10 | F | Y9 | 7 | 0.16 | 0.54 | 11.19 |
| Tr | P11 | M | Y8 M7 | 8 | 1.26 | 2.17 | 14.24 |
| Tr | P12 | F | n.d | n.d | n.d | n.d | n.d |
| Tr | P13 | F | Y41 M2 | 7 | 0.14 | 0.21 | 4.03 |
| Tr | P14 | F | Y26 M8 | 7 | 0.12 | 0.13 | 9.30 |

^†^ Applied reference range is dependent on age and gender. In-house reference ranges were used.

Values are given in mmol/mol creatinine. Abbreviations: 4-OH-Pro: 4-hydroxyproline; D and number: age in days; F: female; M: male: M and number: age in months; n.d: not determined; Pro: proline; Tr: treated; Untr: untreated; Y and number: age in years.

**References:**

[1] Mueller P, Schulze A, Schindler I, Ethofer T, Buehrdel P, Ceglarek U. Validation of an ESI-MS/MS screening method for acylcarnitine profiling in urine specimens of neonates, children, adolescents and adults. *Clin Chim Acta*. 2003;327:47–57.
