# Supplementary material for: Clinical, biochemical, and genetic spectrum of MADD in a South African cohort: an ICGNMD study
Source: Orphanet J Rare Dis. 2024 Jan 14;19:15. doi: 10.1186/s13023-023-03014-8 (PMC10789041; doi:10.1186/s13023-023-03014-8)
Supplement: Supplementary file 3 — Additional file 3. Additional Structural Information. [file 13023_2023_3014_MOESM3_ESM.docx]

**Additional file 3: Additional Structural Information**

**
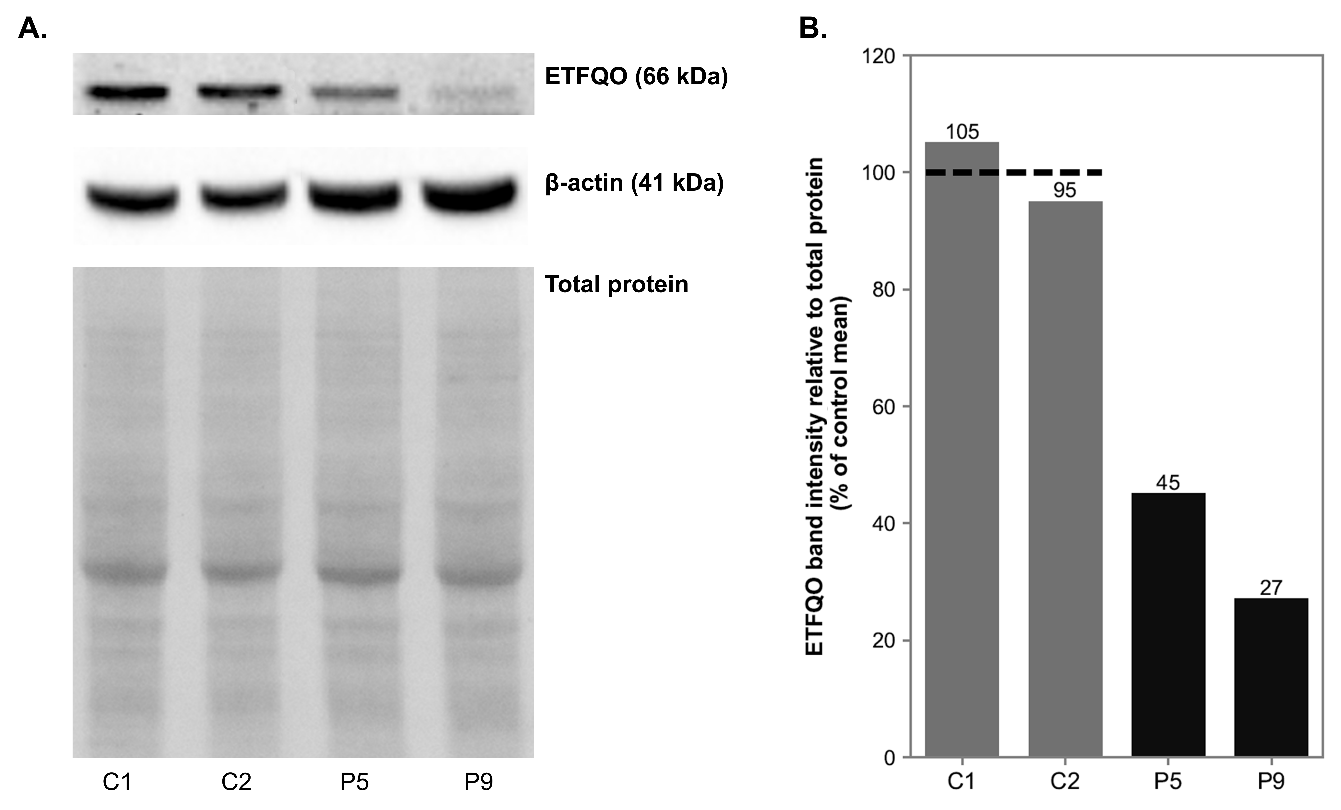
**

**Figure S1.a–b** Structural instability of ETFQO in skin fibroblasts of P5 and P9

(a) Whole cell lysates, prepared from primary skin fibroblasts of P5 (c.[1448C>T];c.[1448C>T]) and P9 (c.[1067G>A];c.[1448C>T]) as well as two healthy controls, C1 and C2 (matched in age, gender, and ethnicity to P9 and P5, respectively), were separated on a 10% SDS-PAGE gel and immunoblotted with primary antibodies against ETFQO (reported weight of the monomeric protein: 64 kDa) and β-actin (42 kDa). (b) Band volume intensities were normalised to the total protein content of each corresponding lane and quantified as a percentage, relative to the mean (100%) of both controls. Abbreviations: ETFQO: electron transfer flavoprotein-ubiquinone oxidoreductase.
